# Analysis and prediction of trends in birth population and HBsAg-positive pregnant women in Zaozhuang City, 2012–2024

**DOI:** 10.3389/fpubh.2026.1933547

**Published:** 2026-10-02

**Authors:** Xiwang Sun, Changling Bian, Jingjing Zhu

**Affiliations:** Zaozhuang Center for Disease Prevention and Control, Zaozhuang, Shandong, China

**Keywords:** birth population, hepatitis B surface antigen, prediction, pregnant women, trend

## Abstract

**Introduction:**

To analyze trends and project future changes in birth population and HBsAg-positive pregnant women in Zaozhuang City from 2012 to 2024, providing baseline data and evidence for optimizing resource allocation and formulating strategies to eliminate mother-to-child transmission (MTCT) of hepatitis B virus (HBV).

**Methods:**

Data on annual births and HBsAg-positive pregnant women were col from the Zaozhuang Statistical Yearbook and the Shandong Provincial Immunization Program Information System (2012 -2024). Birth rate and maternal HBsAg prevalence were calculated. Trends were analyzed using Joinpoint regression to estimate annual percent change (APC) and average annual percent change (AAPC). Curve regression models were fitted to project 2025 levels. Time series analysis based on monthly data was performed for forecasting.

**Results:**

The birth rate declined significantly overall (AAPC = −6.676%, *p* = 0.015), ranging from 5.989‰ to 17.383‰ during 2012 -2024. A period of rapid decline occurred from 2017 to 2021 (APC = −18.730%, p = 0.018). The mean maternal HBsAg prevalence was 2.896%. A significant decreasing trend was observed from 2015 to 2024 (APC = −4.442%, *p* = 0.001). The birth rate and maternal HBsAg prevalence were best fitted by exponential and quadratic function models, respectively, yielding 2025 projections of 5.876‰ and 1.755%. ARIMA models were selected as optifor forecasting monthly time series: ARIMA(0,1,0)(0,1,2)₁₂ for birth count and ARIMA(0,1,1)(1,1,1)₁₂ for HBsAg-positive pregnant women count.

**Conclusion:**

Fertility levels and maternal HBsAg prevalence in Zaozhuang City exhibit sustained declining trends. The fitted model can be used for short-term prediction, providing robust evidence to inform regional healthcare resource planning and the development of comprehensive interventions to eliminate HBV MTCT.

## 1 Introduction

Between 2012 and 2024, Chinese fertility policy underwent adjustments including the “selective two-child,” “universal two-child,” and “three-child” policies (1). Birth population has shown dynamic fluctuations influenced by policy, economic, and social factors, directly affecting resource demands for maternal and child health services. China has a large hepatitis B virus (HBV) carrier population, and achieving the goal of eliminating hepatitis B as a public health threat by 2030 remains a major challenge. Mother-to-child transmission (MTCT) is the most important route (2). Without effective intervention, neonates born to hepatitis B surface antigen (HBsAg)-positive mothers have an infection risk of approximately 90% (3). Tracking the trend of HBsAg-positive pregnant women is a key basis for evaluating the effectiveness of HBV MTCT interruption measures and a necessary action to help achieve the WHO goal. Analyzing trends in birth population and HBsAg-positive pregnant women can predict future prevention and control needs, revealing directions for optimizing maternal and child health and public health resource allocation, such as supply of hepatitis B vaccine and hepatitis B immunoglobulin, follow-up management of key populations, and vaccination service provision. This study analyzes and predicts the trends of birth rate, maternal HBsAg prevalence, number of births, and number of HBsAg-positive pregnant women in Zaozhuang City from 2012 to 2024, providing baseline data and a reference for optimizing maternal and child health services, resource allocation, regional health planning, and preventing and eliminating MTCT risks.

## 2 Data and methods

### 2.1 Data sources

Population data were obtained from the Zaozhuang Statistical Yearbook and the Shandong Provincial Immunization Program Information System. Data on annual births and HBsAg-positive pregnant women were derived from the Shandong Provincial Immunization Program Information System. Data on the number of live births by year and month in Zaozhuang City, along with the results of serum hepatitis B surface antigen (HBsAg) testing performed during pregnancy or at antenatal screening for each corresponding year and month, were obtained from the Shandong Provincial Immunization Program Information System. Both the total birth counts and the number of HBsAg-positive parturient women were derived from this system. In the present study, only HBsAg-positive parturient women were included in the analysis.

### 2.2 Methods

#### 2.2.1 Joinpoint regression analysis

Based on annual data of birth rate and maternal HBsAg prevalence from 2012 to 2024, Joinpoint regression was used to evaluate temporal trends. The permutation test (Monte Carlo simulation) in Joinpoint software was applied to determine optimal joinpoints, dividing the time series into intervals. Annual percent change (APC) and average annual percent change (AAPC) were calculated. APC > 0 indicates an upward trend, APC < 0 a downward trend. AAPC reflects the overall trend. Statistical significance was determined using 95% confidence intervals (CI), *t*-tests, and *p*-values.

#### 2.2.2 Curve regression

Curve regression can reveal nonlinear relationships through flexible functional forms. Based on the distribution characteristics of birth rate and maternal HBsAg prevalence from 2012 to 2024, curve regression (linear, quadratic, cubic, power, exponential, logistic) was performed. The optimal model was selected based on adjusted *R*^2^, *t*-test and *F*-test results.

#### 2.2.3 Time series analysis

Time series datasets were constructed for monthly birth counts and monthly HBsAg-positive pregnant women from January 2012 to December 2024. The stationarity of the model is assessed through the construction of time-series plots and the application of unit root tests. Based on autocorrelation function (ACF) and partial autocorrelation function (PACF) plots, autoregressive integrated moving average (ARIMA) models were established.

For parameter optimization of the ARIMA model in this study, we adopted the normalized Bayesian information criterion (nBIC) as the optimization metric, supplemented by visual inspection of the ACF and PACF plots, and applied the Ljung–Box Q test to the model residuals. For the non-seasonal orders (*p, d, q*), the search ranges for p and q were set from 0 to 2, and the optimal differencing order d was determined by examining the time series plot. For the seasonal orders (P, D, Q) m, given the data periodicity (m = 12 for monthly data), we performed a grid search over P and Q within the same range of 0 to 2, and selected the seasonal differencing order D based on the ACF and PACF plots.

Among the generated candidate models, the final model was chosen by prioritizing those with a relatively lower nBIC value and ensuring that the residuals exhibited white noise behavior. The final models were used to forecast data from January to June 2025, and prediction performance was evaluated.

### 2.3 Statistical analysis

Joinpoint Regression Program 5.0.2 was used to identify joinpoints and perform trend analysis. Excel 2021, SPSS 26.0 and EViews 13.0 were used for data processing and analysis. The significance level was *α* = 0.05.

## 3 Results

### 3.1 Basic characteristics

From 2012 to 2024, a total of 608,559 children were born in Zaozhuang City, and 17,627 HBsAg-positive pregnant women were recorded. Annual birth rate ranged from 5.989‰ to 17.383‰ (2023 vs. 2017), with an average annual birth rate of 11.537‰. Annual maternal HBsAg positivity rate ranged from 2.027 to 3.306% (2024 vs. 2015), with an average annual prevalence of 2.896% (see Figure 1).

**Figure 1:**
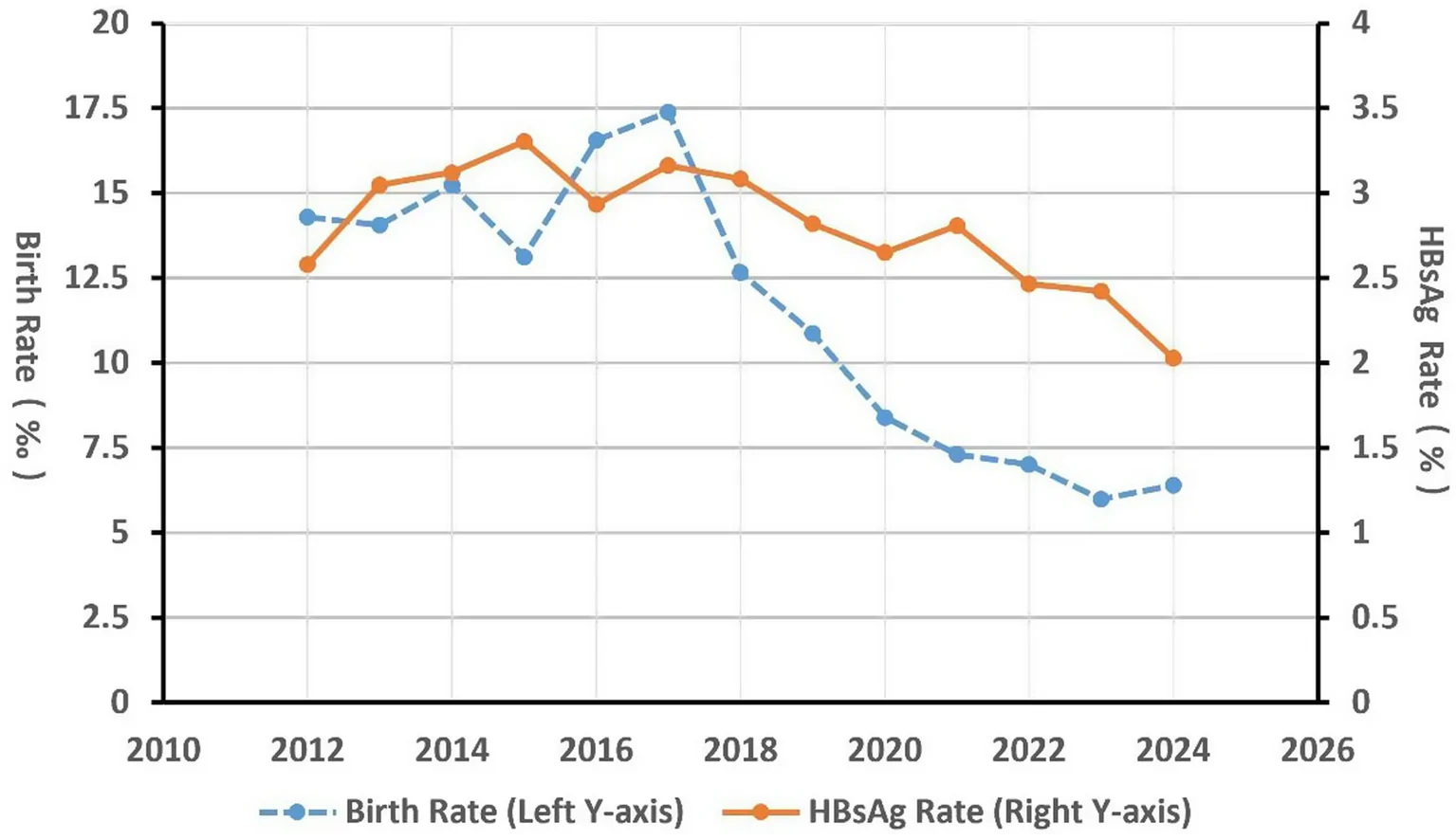
Trends in birth rate and maternal HBsAg positivity rate in Zaozhuang City, 2012–2024.

### 3.2 Trends in birth rate and maternal HBsAg prevalence

Joinpoint regression showed that overall birth rate from 2012 to 2024 exhibited a significant downward trend (AAPC = −6.676, 95%*CI*: −11.371% to −1.309%, t = −2.440, *p* = 0.015). For maternal HBsAg prevalence from 2012 to 2024, no significant overall trend was observed (AAPC = −1.464, 95%*CI*: −3.984 to 1.123%, *t* = −1.115, *p* = 0.265).

Two joinpoints were identified for birth rate (2017 and 2021). From 2012 to 2017, APC = 3.352% (*p* = 0.275); from 2021 to 2024, APC = −4.516% (*p* = 0.477); from 2017 to 2021, a rapid downward trend was observed (APC = −18.730%, *p* = 0.018). For maternal HBsAg prevalence, one joinpoint was identified in 2015. From 2012 to 2015, APC = 8.039% (*p* = 0.134); from 2015 to 2024, a significant downward trend was observed (APC = −4.442%, *p* = 0.001) (see Table 1).

**Table 1:** Joinpoint regression analysis of birth rate and maternal HBsAg positivity rate in Zaozhuang City, 2012–2024.

Indicator	Period	APC (%)	95%CI (%)	*t*-value	*p*-value
Birth rate	2012–2017	3.352	−3.548 ~ 10.745	1.227	0.275
	2017–2021	−18.730	−30.364 ~ −5.153	−3.451	0.018
	2021–2024	−4.516	−18.185 ~ 11.437	−0.769	0.477
Maternal HBsAg rate	2012–2015	8.039	−2.919 ~ 20.233	1.667	0.134
	2015–2024	−4.442	−6.289 ~ −2.558	−5.366	0.001

### 3.3 Curve fitting and prediction of birth rate and maternal HBsAg prevalence

We performed curve fitting for birth rate and maternal HBsAg positivity rate using linear, quadratic, cubic, power, exponential, and logistic regression models. The optimal model for each outcome was selected by comprehensive consideration of the adjusted *R*^2^, the *F*-test and associated *p*-values, and the width of prediction intervals. The final fitted models were: for birth rate, 𝑦_1_ = 19.755𝑒^−0.087𝑡^(∆𝑅^2^ = 0.755, *F* = 38.016, *p* < 0.001); and for maternal HBsAg positivity rate, 𝑦_2_ = −0.017𝑡^2^ + 0.180𝑡 + 2.636(∆𝑅^2^ = 0.809, *F* = 26.459, *p* < 0.001) (see Table 2).

**Table 2:** Model fitting results of birth rate and maternal HBsAg positivity rate in Zaozhuang City.

Dependent variable	Model type	Variable	Coefficient	*t*-value	*p*-value
Birth rate	Exponential	*t*	−0.087	−6.166	<0.001
		Constant	19.755	8.969	<0.001
Maternal HBsAg positivity rate	Quadratic	*t*	0.180	3.539	0.005
		*_t_2*	−0.017	−4.909	0.001
		Constant	2.636	17.026	<0.001

Based on these fitted models, the predicted values (with 95% prediction intervals) for 2025 were 5.876‰ (3.622‰–9.534‰) for birth rate and 1.755% (1.262–2.248%) for maternal HBsAg positivity rate.

### 3.4 Time series analysis of monthly birth counts and HBsAg-positive pregnant women

#### 3.4.1 Model fitting

Time series were constructed for the monthly counts of births and HBsAg-positive parturient counts over the period from January 2012 to December 2024, and time-series analysis and forecasting were subsequently performed.

Together with the time-series plots (Figures 2A,B), unit root tests applied to the birth counts and HBsAg-positive maternal counts gave *t*-values of −1.109 (*p* = 0.712) and −0.976 (*p* = 0.761), respectively, suggesting that both series are non-stationary. Based on ACF/PACF plots, differencing and parameter tuning were performed.

**Figure 2:**
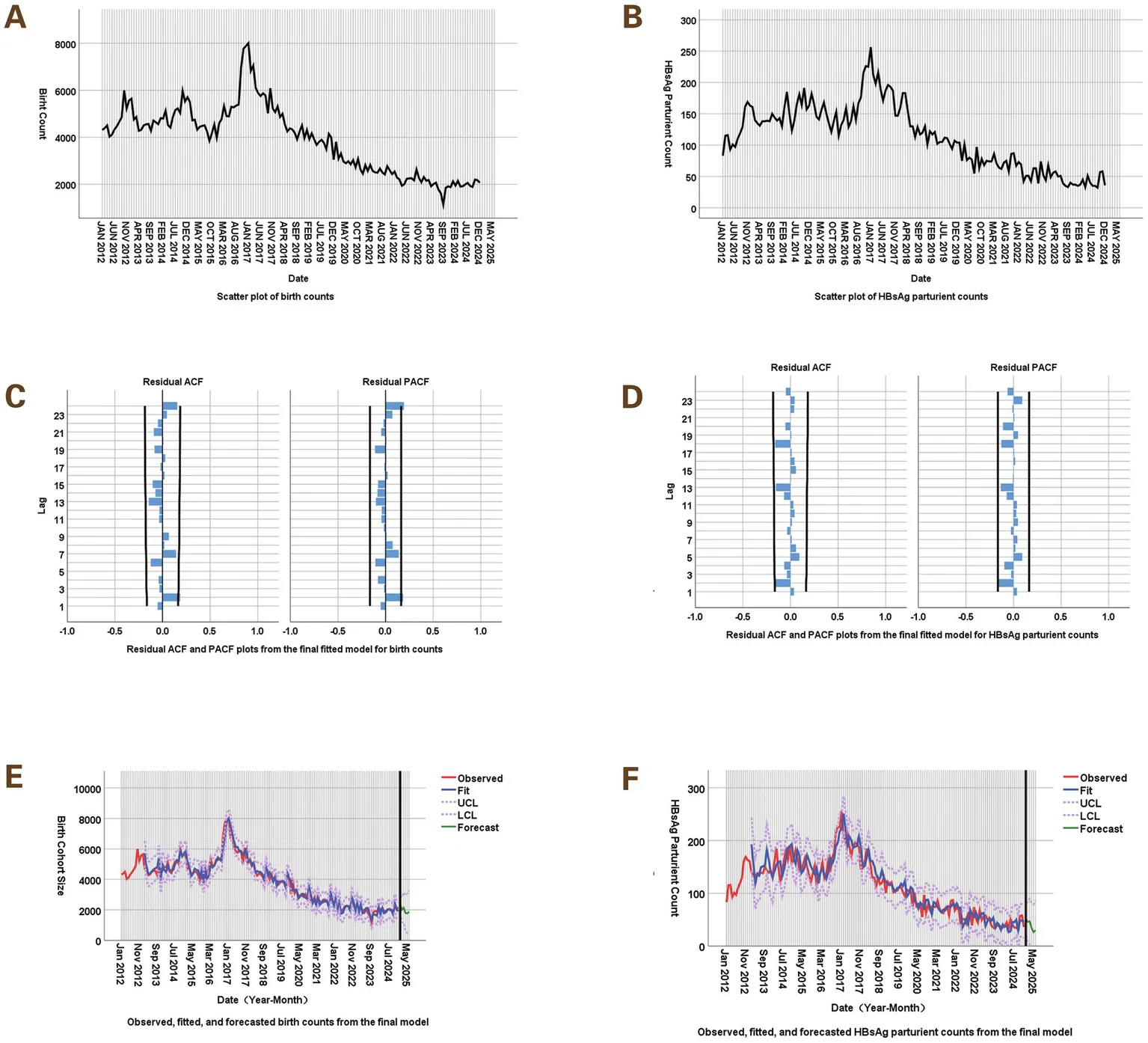
Time series plots, residual ACF and PACF plots, and forecast plots for birth numbers and maternal HBsAg-positive cases in Zaozhuang City. **(A)** Scatter plot of birth counts. **(B)** Scatter plot of HBsAg parturient counts. **(C)** Residual ACF and PACF plots from the final fitted model for birth counts. **(D)** Residual ACF and PACF plots from the final fitted model for HBsAg parturient counts. **(E)** Observed, fitted, and forecasted birth counts from the final model. **(F)** Observed, fitted, and forecasted HBsAg parturient counts from the final model.

The optimal model for birth counts was ARIMA(0,1,0)(0,1,2)12 (Ljung-Box Q = 18.723, *p* = 0.283, *R*^2^ = 0.955, *RMSE* = 313.408, *MAPE* = 6.379, *MAE* = 226.139, nBIC = 11.564); The optimal model for HBsAg-positive pregnant women was.

ARIMA(0,1,1)(1,1,1)12 Ljung-Box Q = 17.457, *p* = 0.292, *R*^2^ = 0.894, *RMSE* = 17.491, *MAPE* = 14.315, *MAE* = 13.632, nBIC = 5.827). Residuals were white noise for both models (see Table 3; Figures 2C,D).

**Table 3:** Model fitting results for number of births and HBsAg-positive parturient women in Zaozhuang City.

Birth counts			HBsAg-positive pregnant women		
Model type	nBIC	White noise	Model type	nBIC	White noise
ARIMA(2,1,1)(1,1,1)12	11.639	Yes	ARIMA(0,1,1)(1,1,0)12	6.039	No
ARIMA(2,1,0)(1,1,1)12	11.596	Yes	ARIMA(0,1,1)(1,1,1)12	5.827	Yes
ARIMA(0,1,2)(1,1,1)12	11.591	Yes	ARIMA(1,1,1)(1,1,1)12	6.061	No
ARIMA(0,1,0)(0,1,2)12	11.564	Yes	ARIMA(2,1,1)(1,1,1)12	6.094	No
ARIMA(2,1,0)(1,0,1)12	11.626	Yes	ARIMA(0,1,2)(1,1,0)12	6.054	No
ARIMA(1,1,2)(1,1,1)12	11.633	Yes	ARIMA(0,1,2)(1,1,1)12	5.846	Yes
ARIMA(0,1,0)(1,1,1)12	11.550	No	ARIMA(0,1,0)(1,1,1)12	5.841	No
ARIMA(1,1,0)(1,1,1)12	11.588	No	ARIMA(2,1,0)(1,1,1)12	5.848	Yes
ARIMA(1,1,1)(1,1,1)12	11.632	Yes	ARIMA(2,1,2)(1,1,1)12	5.918	Yes

#### 3.4.2 Model prediction performance

The fitted models showed good performance. Forecasts for January–June 2025 gave mean absolute percentage errors of 17.47% for birth counts and 15.84% for HBsAg-positive pregnant women (see Figures 2E,F; Table 4).

**Table 4:** Model fitting performance for birth numbers and maternal HBsAg-positive cases.

Time (2025)	Birth counts				HBsAg-positive pregnant women			
	Actual	Forecast	Absolute error	Relative error (%)	Actual	Forecast	Absolute error	Relative error (%)
January	2044	2,360	316	15.46	56	51	5	8.93
February	1,642	2004	362	22.05	34	46	12	35.29
March	1798	2,141	343	19.08	33	47	14	42.42
April	1,562	1793	231	14.79	32	34	2	6.25
May	1,535	1762	227	14.79	22	26	4	18.18
June	1,578	1874	296	18.76	25	30	5	20.00
Total/Mean	10,159	11,934	1775	17.47	202	234	32	15.84

## 4 Discussion

Zaozhuang City implemented the “selective two-child,” “universal two-child,” and “three-child” policies in 2014, 2016, and 2021, respectively. After the “selective two-child” policy, the birth rate did not increase in 2015 but decreased by 13.84% compared to the previous year. Following the “universal two-child” policy, the birth rate increased by 26.11% in 2017, reaching the highest level of the period, possibly due to the concentrated release of fertility demand among women of childbearing age in the early stage of policy relaxation. The birth rate declined from 17.38‰ in 2017 to 5.99‰ in 2023, showing a downward trend for seven consecutive years. After the “three-child” policy, no increase or fluctuation in birth rate was observed. From 2017 to 2021, the birth rate declined rapidly at approximately 18.73% per year. In 2024, the birth rate was 6.41‰, ending the seven-year consecutive decline since 2017. This change may be attributed to two factors: post-COVID catch-up births and the zodiac preference effect (especially the Dragon year fertility preference). Some scholars believe this rebound may be temporary (4, 5). In this study, based on the birth rate series, we established a model to predict that the birth rate in Zaozhuang City in 2025 will be 5.88‰, which is lower than the 2024 level and also below the 2023 figure of 5.99‰. This suggests the end of the short-term rebound, which may be attributable to multiple factors, including changes in fertility attitudes and intentions, economic conditions, and the continuing trend of delayed marriage, among others (6, 7). The projected continued decline in the birth rate in 2025 indicates that the overall future demand for public health resources for newborns will continue to decrease, which may provide a reference for resource demand forecasting and rational planning (8).

The average annual maternal HBsAg prevalence in Zaozhuang City was 2.896%, slightly higher than that of Jinan City (2.84%) (9), lower than that of Jiangsu Province (3.05%) (10), and lower than the national prevalence level (6.17%) (11). Maternal HBsAg prevalence decreased from 3.31% in 2015 to 2.07% in 2024, with an average annual decline rate of approximately 4.44%, showing a year-by-year downward trend consistent with trends in Jinan (9) and nationally (12). The prediction shows that the maternal HBsAg prevalence in Zaozhuang City in 2025 will be 1.76%, continuing the downward trend and remaining at a medium endemic level (13). This is related to the natural decline in the infected base and proportion among women of childbearing age after the universal vaccination of hepatitis B vaccine (14).

The optimal time series models were ARIMA(0,1,0)(0,1,2)12 for monthly birth counts and ARIMA(0,1,1)(1,1,1)12 for monthly HBsAg-positive pregnant women. Inspection of the residual ACF and PACF plots (Figures 2C,D), together with the Ljung–Box test results (all *p* > 0.05), revealed that no significant autocorrelation remained; this indicated that the residuals of the fitted model were white noise. The fitted curves closely tracked the long-term trend and the monthly fluctuation turning points of the original observed series. Most observed values lay within the 95% confidence intervals for the majority of time points (Figures 2E,F), suggesting that the model successfully captured the main dynamics of the series. These models are suitable for forecasting monthly numbers of births and HBsAg-positive pregnant women in the coming months. The overestimation of actual values by the model’s monthly predictions may be attributable to the model’s inability to detect inflection points promptly, as it simply extrapolates the historical trend, potentially resulting in upward bias. In practice, new observations can be incorporated to retrain the models for improved prediction (15). Based on the predicted values from the fitted models for birth rate and birth counts, we can optimize the allocation of healthcare resources for specific populations, such as rational allocation of hepatitis B vaccine doses, optimal allocation of delivery beds, obstetricians, midwives, and vaccination units. Based on the fitted models, we can predict the number of HBsAg-positive pregnant women in the coming months and, combined with the predicted maternal HBsAg prevalence, moderately reduce the procurement and stockpiling of hepatitis B vaccine and hepatitis B immunoglobulin, among others. For neonates born to HBsAg-positive mothers, the combined immunoprophylaxis strategy (hepatitis B vaccine + hepatitis B immunoglobulin) has greatly reduced the MTCT rate (14, 16, 17), but there remains a risk of MTCT (18, 19). Postpartum management and monitoring, follow-up management of neonatal vaccination, and health education still need to be strengthened to better consolidate the effect of hepatitis B prevention and control (20).

The study data did not include information on maternal HBeAg, viral load, etc., which limits the analysis of HBV MTCT risk. The exponential model for birth rate and the quadratic function model for maternal HBsAg prevalence are only suitable for short-term predictions; long-term predictions often deviate from actual patterns and are not applicable. The prediction models did not account for future policy changes (e.g., new fertility incentive measures).

Against the background of low birth rate and a year-by-year decreasing trend in maternal HBsAg prevalence in Zaozhuang City, to better eliminate HBV transmission risk, we can optimize the prevention and treatment system, integrate public health and medical services, and establish a comprehensive prevention and control system covering the entire life cycle of HBV infection, including prevention, screening, diagnosis, and treatment (21, 22). Integration of medical and preventive services and establishment of a public health management model (23) can reduce the disease burden on patients. Based on the results of this study, data support and a reference can be provided for optimizing regional healthcare resource allocation and establishing a “data-driven, regionally coordinated, key population-covered” three-dimensional prevention and control network.

## 5 Conclusion

The concomitant decline in both the birth rate and the maternal HBsAg positivity rate in Zaozhuang City reflects positive developments in the region’s demographic transition and its hepatitis B prevention and control efforts. This dual downtrend not only provides a favorable epidemiological context for accelerating the elimination of mother-to-child transmission (EMTCT) of hepatitis B but also imposes heightened requirements for the refined management of immunization program supplies and the continuous improvement of service quality. It is recommended that local health authorities establish an annual rolling prediction framework based on the aforementioned model, incorporating dynamic surveillance data on birth populations and HBsAg positivity rates into the decision-making processes for immunization program planning. This approach would enable the scientific and precise formulation of procurement plans for vaccines and hepatitis B immunoglobulin (HBIG), thereby ensuring that the quality and standards of EMTCT services remain uncompromised despite the overall decline in the neonatal population.

## Data Availability

The original contributions presented in the study are included in the article/supplementary material, further inquiries can be directed to the corresponding authors.
